# Supplementary material for: In silico generation of synthetic cancer genomes using generative AI
Source: Cell Genom. 2025 Aug 12;5(11):100969. doi: 10.1016/j.xgen.2025.100969 (PMC12648103; doi:10.1016/j.xgen.2025.100969)
Supplement: Document S1. Figures S1–S22 [file mmc1.pdf]

Cell Genomics, Volume *5*

Supplemental information

In silico generation of synthetic cancer genomes using generative AI

Ander Díaz-Navarro, Xindi Zhang, Wei Jiao, Bo Wang, and Lincoln Stein

```
//------------------------------------------------------------------------
// Initialization
//------------------------------------------------------------------------

LOAD refGenome
SET nDonors, tumorType and onlyMuts
LOAD models USING tumorType

//------------------------------------------------------------------------
// Simulate global cohort data
//------------------------------------------------------------------------

GENERATE cohortCharacteristics USING models and nDonors
DETERMINE cohortVAF USING models and nDonors
IF NOT onlyMuts THEN
  GENERATE cohortCNA_SV USING models, nDonors
                              and cohortCharacteristics
END IF

//------------------------------------------------------------------------
// Simulate specific case data
//------------------------------------------------------------------------

FOR each donorID in nDonors
  GET donorCharacteristics FROM cohortCharacteristics[donorID]
  GET donorVAF FROM cohortVAF[donorID]

  DETERMINE donorSex USING tumorType

  GENERATE mutDrivers USING models
  FOR mutationType in donorCharacteristics
    GENERATE mutations USING models, mutationType and donorSex
    STORE mutations in mutContexts
  END FOR

  COMPUTE totalMutations as length of mutContexts and donorDrivers
  GENERATE mutCoordinates USING models, totalMutations and donorSex
  ASSIGN mutCoordinates TO refGenome USING mutContexts

  IF onlyMuts THEN
    DETERMINE mutVAFs USING models, donorVAF and totalMutations
    PRINT donorVCF USING donorID, mutContexts, mutCoordinates,
                         mutDrivers and mutVAFs
  ELSE
    DETERMINE donorVCF USING donorID, mutContexts,
                             mutCoordinates and mutDrivers

    GET donorCNA_SV FROM cohortCNA_SV[donorID]
    DETERMINE driverCNA USING models and donorSex
    GENERATE mutCNA USING models, donorCNA_SV and donorSex
    GENERATE mutSV USING models, donorCNA_SV and donorSex
    ALIGN mutCNA, driverCNA, and mutSV

    DETERMINE eventOrder USING donorVCF, mutCNA and mutSV
    COMPUTE mutVAFs USING eventOrder
    UPDATE donorVCF USING mutVAFs

    PRINT donorVCF, mutCNA, mutSV and eventOrder
    PLOT mutCNA and mutSV
  END IF
END FOR
```

cohortCharacteristics (CTAB-GAN+ model-1)

	del	ins	dnp	tnp	sbs1	sbs2	…	total
sim1	20	34	4	1	359	102	…	1058
sim2	75	91	14	4	1053	394	…	3619

cohortVAF (sampling)

	sim1	sim2	…
meanVAF	0.34	0.41	…

guides the simulation

cohortCNA_SV (CTAB-GAN+ model-2)

	tra	h2hinv	t2tinv	cna	cna_len	total
sim1	4	1	3	7	19.05	1058
sim2	21	7	19	41	84.05	3619

mutDrivers (CTAB-GAN+ model-3)

driver1	driver2	driver3	…
1	0	2	…

mutContexts (CTAB-GAN+ model-4)

len	signature	mut	r.ctx	a.ctx
-38	.	del	GGG	...
-1	.	del	GCC	...
-1	.	del	ATT	...
…	…	…	…	…
**0**	**sbs5**	**snp**	**GTT**	**GAT**
0	sbs5	snp	AGA	ACA
0	sbs5	snp	TCC	TTC

mutCoordinates (TVAE)

```
98308532 222203069
77667785 69818515  …
```

by chromosome cumulative length

100bp window

AAGGGTTTTGTGACTC**GTT**TGTAAGAAGTCATACGGAAGTCGCTTCCTGG

assigned position

mutVAFs (sampling)

```
0.34 -> 0.3 0.4 0.3 …
```

donorVCF

#CHROM	POS	ID	REF	ALT	QUAL	FILTER	INFO
1	15722779	sim1	C	T	.	.	AF=0.3;MS=SBS5
1	21607949	sim1	T	A	.	.	AF=0.4;MS=SBS5
…	…	…	…	…	…	…	…
Y	7034501	sim1	G	A	.	.	AF=0.3;MS=SBS1
Y	8468797	sim1	T	G	.	.	AF=0.28;MS=SBS5

mutCNA (CTAB-GAN+ model-5)

chrom	start	end	major_cn	minor_cn	donor_id	study	cna_id
1	1	249250621	1	1	sim1	*	cna0
2	1	243199373	1	1	sim1	*	cna1

mutSV (CTAB-GAN+ model-6)

chrom1	start1	end1	chrom2	start2	end2	strand1	strand2	svclass	cna_id	sv_id	allele	donor_id	study
5	1	2	5	46100000	46100001	-	+	DUP	cna4	sv0	major	sim1	*
5	180623559	180623560	5	180915260	180915261	-	+	DUP	cna6	sv1	major	sim1	*

eventOrder

index	class	cna_id	major_minor	event_id	allele	from_allele	to_allele
0	MUT	no_cna	.	snv267	allele_2_major	.	.
1	MUT	no_cna	.	snv496	allele_2_major	.	.

donorVCF

#CHROM	POS	ID	REF	ALT	QUAL	FILTER	INFO
1	15722779	sim1	C	T	.	.	AF=0.3;MS=SBS5;TA=2;AL=allele_2_major;CN=no_cna
1	21607949	sim1	T	A	.	.	AF=0.4;MS=SBS5;TA=2;AL=allele_1_minor;CN=no_cna
…	…	…	…	…	…	…	…
Y	7034501	sim1	G	A	.	.	AF=0.3;MS=SBS1;TA=2;AL=allele_1_minor;CN=no_cna
Y	8468797	sim1	T	G	.	.	AF=0.28;MS=SBS5;TA=2;AL=allele_2_major;CN=no_cna

Figure S1. Pseudocode overview of the main steps in the OncoGAN pipeline, illustrating the output of each model and their connections, related to Figure 1.

Briefly, models specific to the user-defined tumor type are first loaded. Based on the requested number of donors, OncoGAN simulates cohort-level characteristics. Subsequently, additional features –such as mutational context and position, variant allele frequency, driver mutations, and CNA-SV events– are generated for each donor.

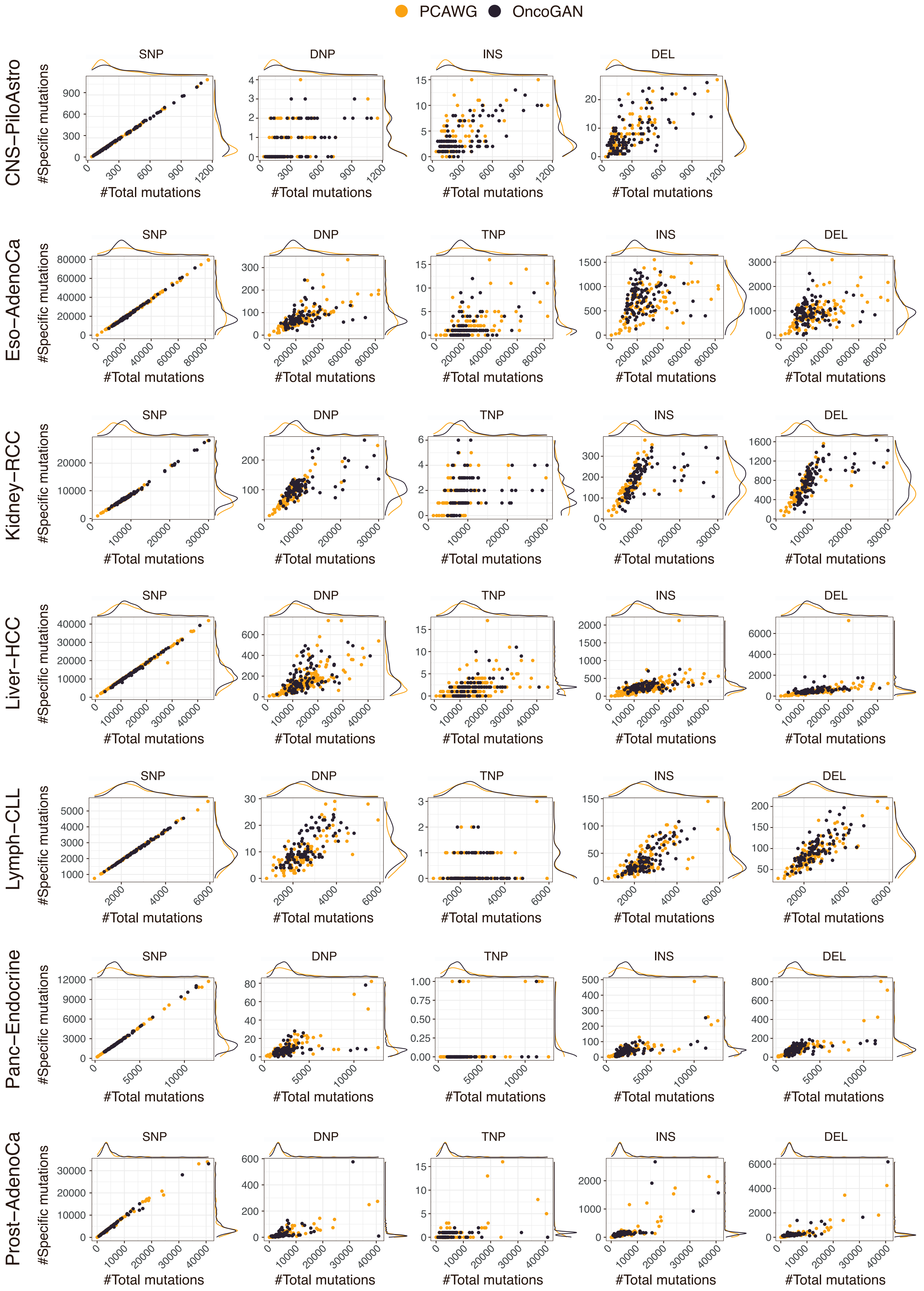

Figure S2. Scatter and density plots comparing the number of specific mutation types to the total number of mutations for each donor, related to Figure 2A.
Real donors from PCAWG are shown in orange, and simulated donors from OncoGAN are shown in black. In all cases, the distributions are very similar, although there are minor challenges in simulating donors with very low mutational burdens in Eso-AdenoCa and Kidney-RCC tumor types.

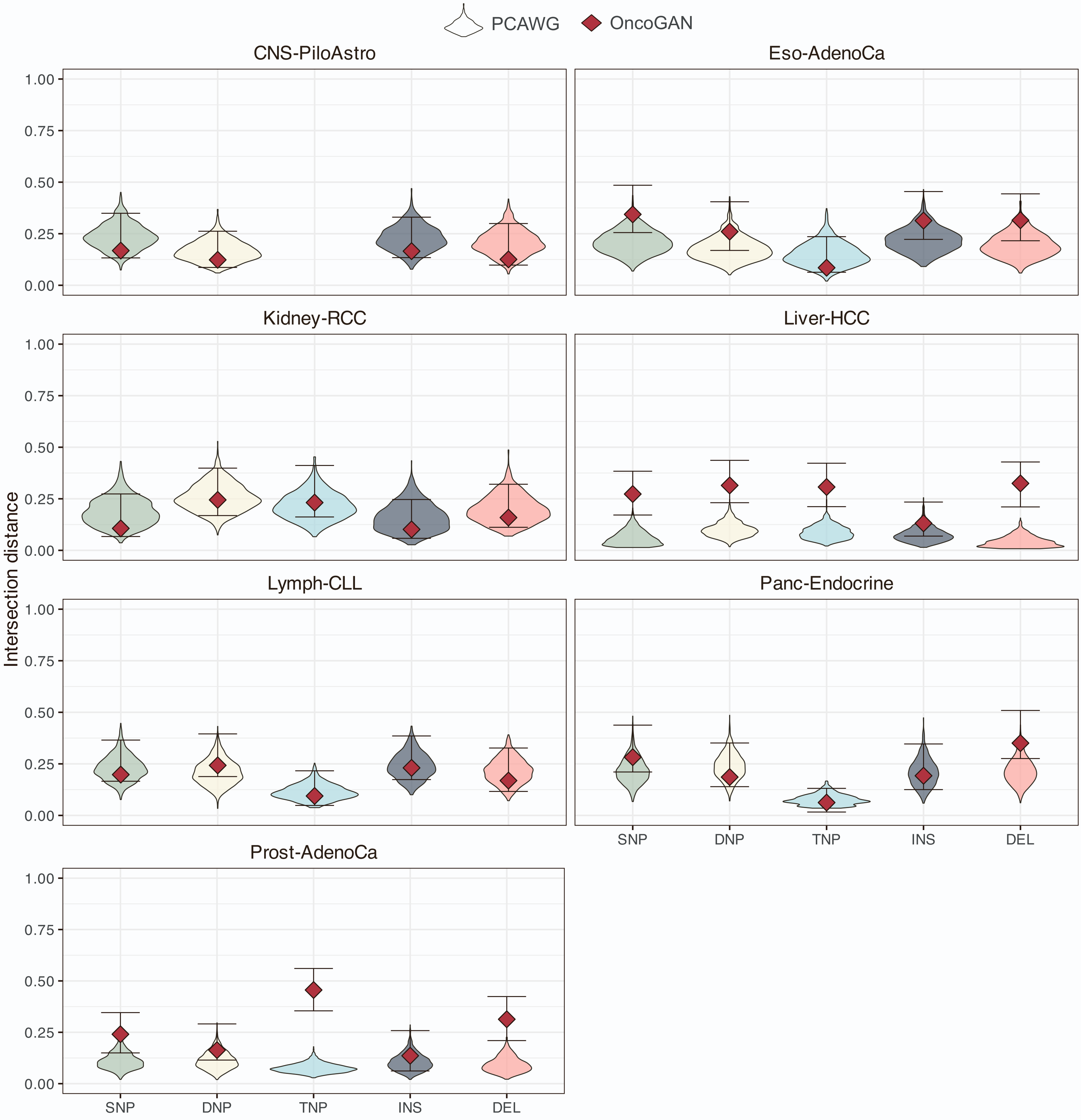

Figure S3. Violin plots illustrating the distribution of intersection distances between two randomly sampled populations from the PCAWG dataset and the scores comparing OncoGAN simulations to the actual dataset, related to Figure 2B.
For most mutation and tumor types, the simulations correspond closely with the obtained results from comparisons between two subpopulations in the real data (1,000 iterations), indicating a high degree of similarity between the PCAWG and OncoGAN distributions. Lower scores indicate greater similarity between the populations. Error bars represent confidence intervals calculated via bootstrapping the OncoGAN dataset (1,000 iterations).

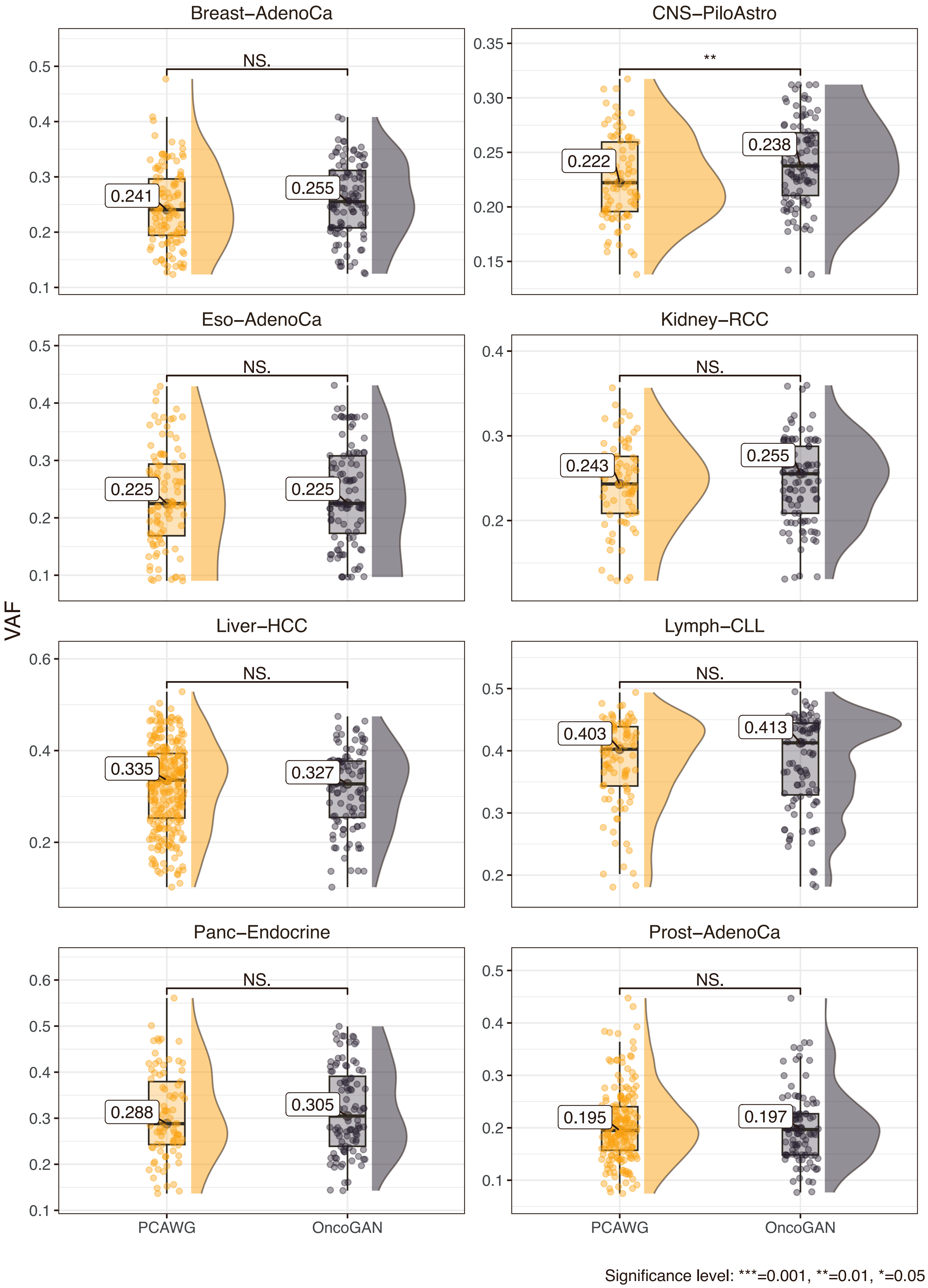

Figure S4. Comparative density and box plots showing donor's distributions of mean variant allele frequency (VAF) across eight tumor types, related to STAR Methods.

Each panel compares the VAFs from real samples (PCAWG) in orange and simulated samples (OncoGAN) in black. Notably, only the CNS-PiloAstro panel shows a statistically significant difference, which may not be biologically relevant as both distributions are very similar with median VAFs of 0.222 and 0.238, respectively. This underscores the accuracy of the OncoGAN simulations in reflecting real data. The Wilcoxon test was used to compare the groups. The sample size used for each comparison corresponds to the number of donors available for each tumor type. NS.: p-value > 0.05; *: p-value <= 0.05; **: p-value <= 0.01; ***: p-value <= 0.001.

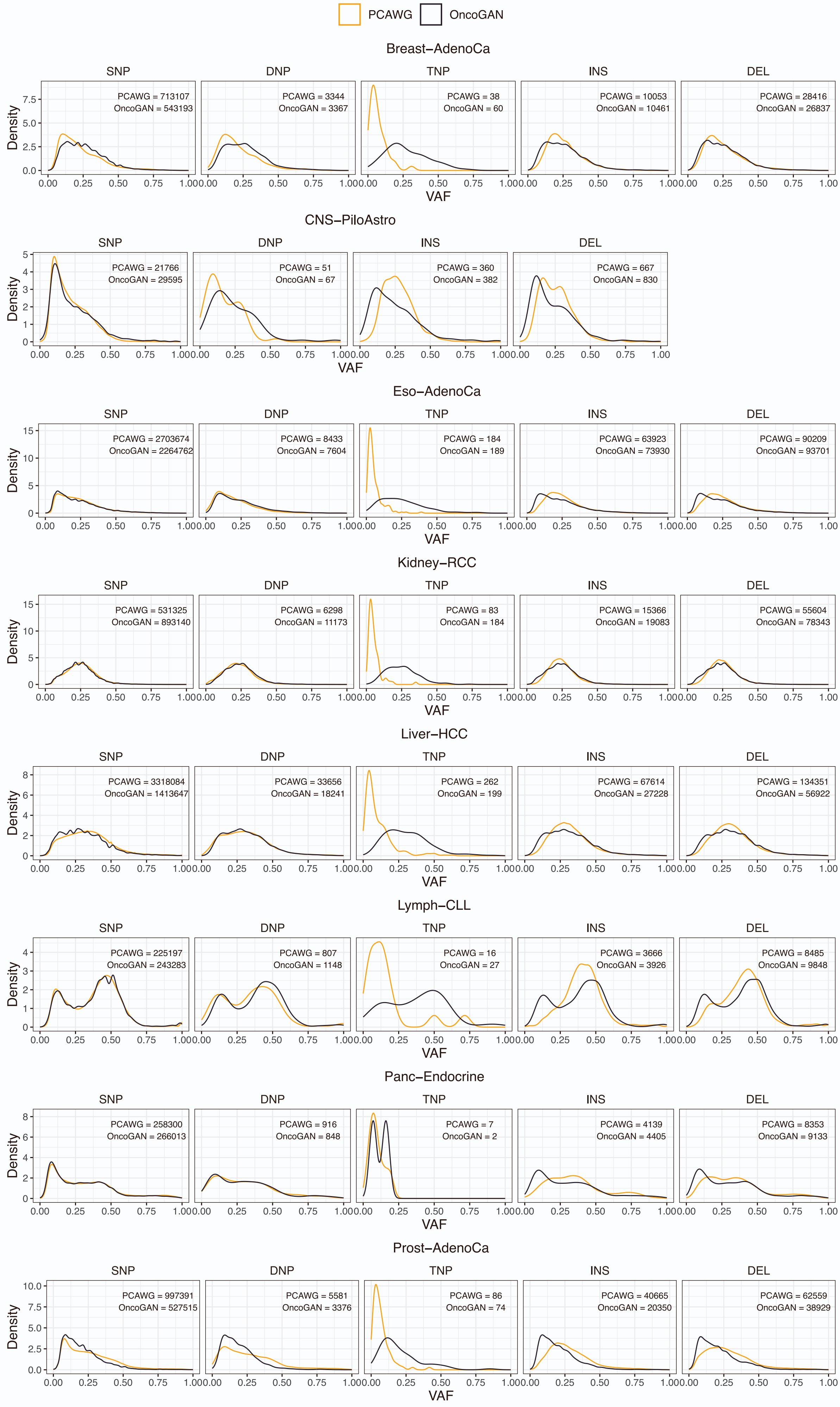

Figure S5. Density plots for the variant allele frequency (VAF) for each type of mutation and tumor, related to STAR Methods.

The densities are very similar between real (orange) and simulated (black) VAFs. The number of each type of mutation in each dataset is also reported. The greatest differences are observed in TNPs due to its low frequency.

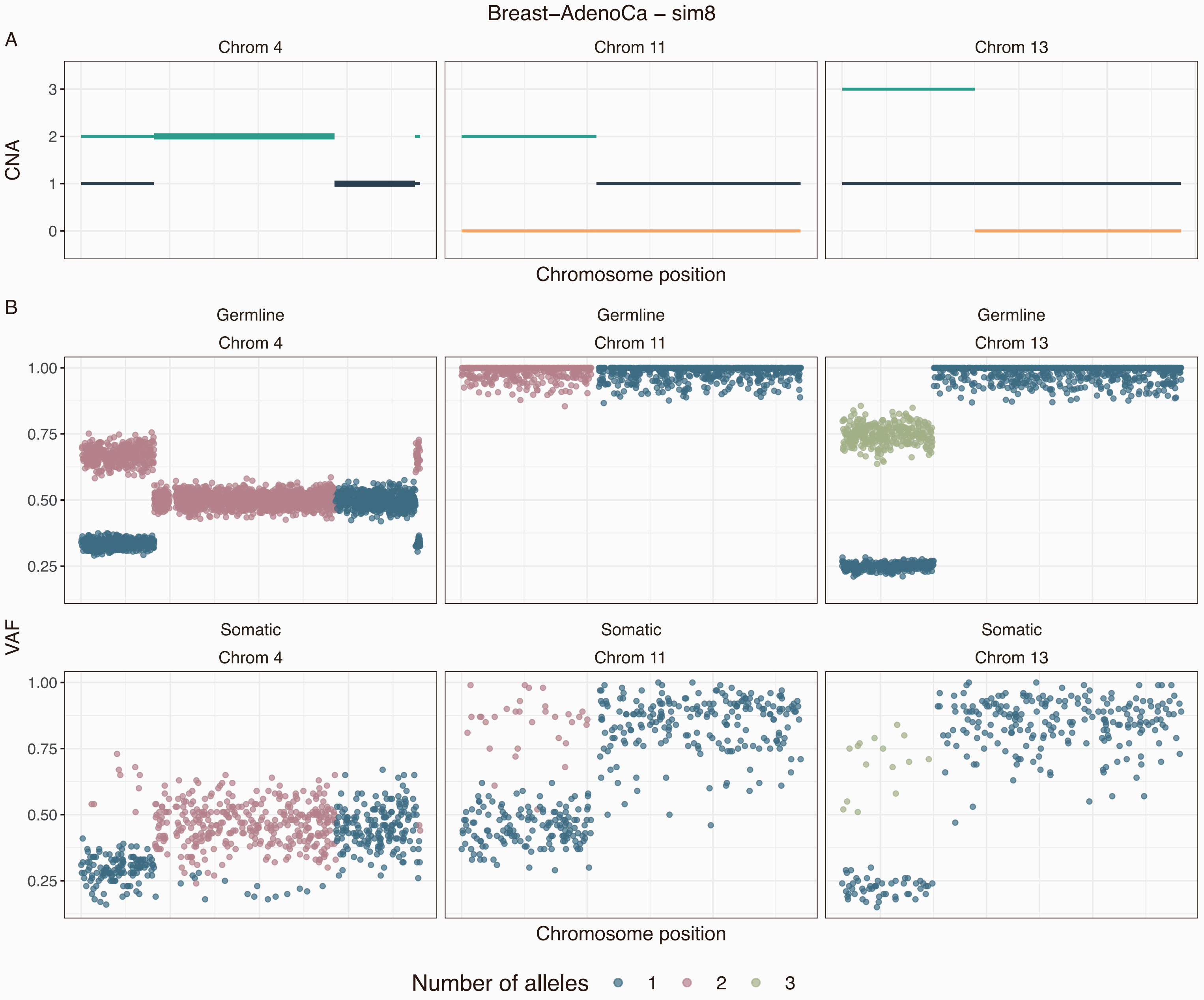

Figure S6. Quality control plots for validating the accuracy of CNA-driven allele frequency simulation, related to STAR Methods.

A) Copy number alteration profiles for three chromosomes, each showing different combinations of copy number events. B) Variant allele frequency distributions of germline and somatic mutations, reflecting the underlying CNA pattern. Points are colored by the number of alleles in which each mutation appears.

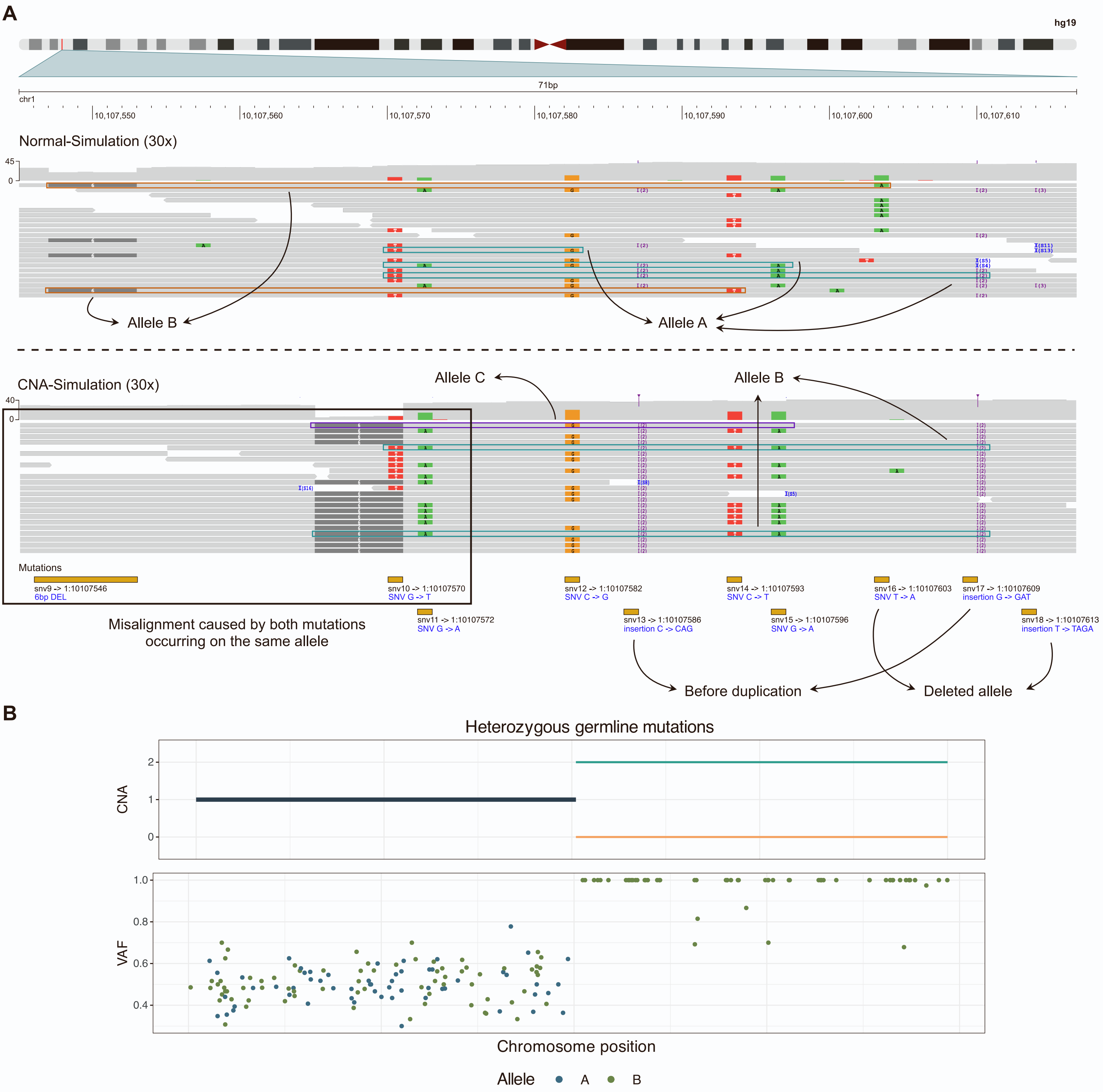

Figure S7. Overview of in silico tumor BAM generation and resulting genomic features, related to STAR Methods. A) Alignment screenshot of the BAM generated by integrating OncoGAN with InSilicoSeq using two approaches: a simpler one, where OncoGAN simulates only a VCF with mutations, and a more complex one that includes both mutations and CNAs. Phased mutations are visible, and variant allele frequency is influenced by copy number status and the order of events. B) CNA profile of the in silico BAM and the variant allele frequency of germline mutations that were heterozygous prior to any tumor event, demonstrating how germline variants reflect the structural variant events.

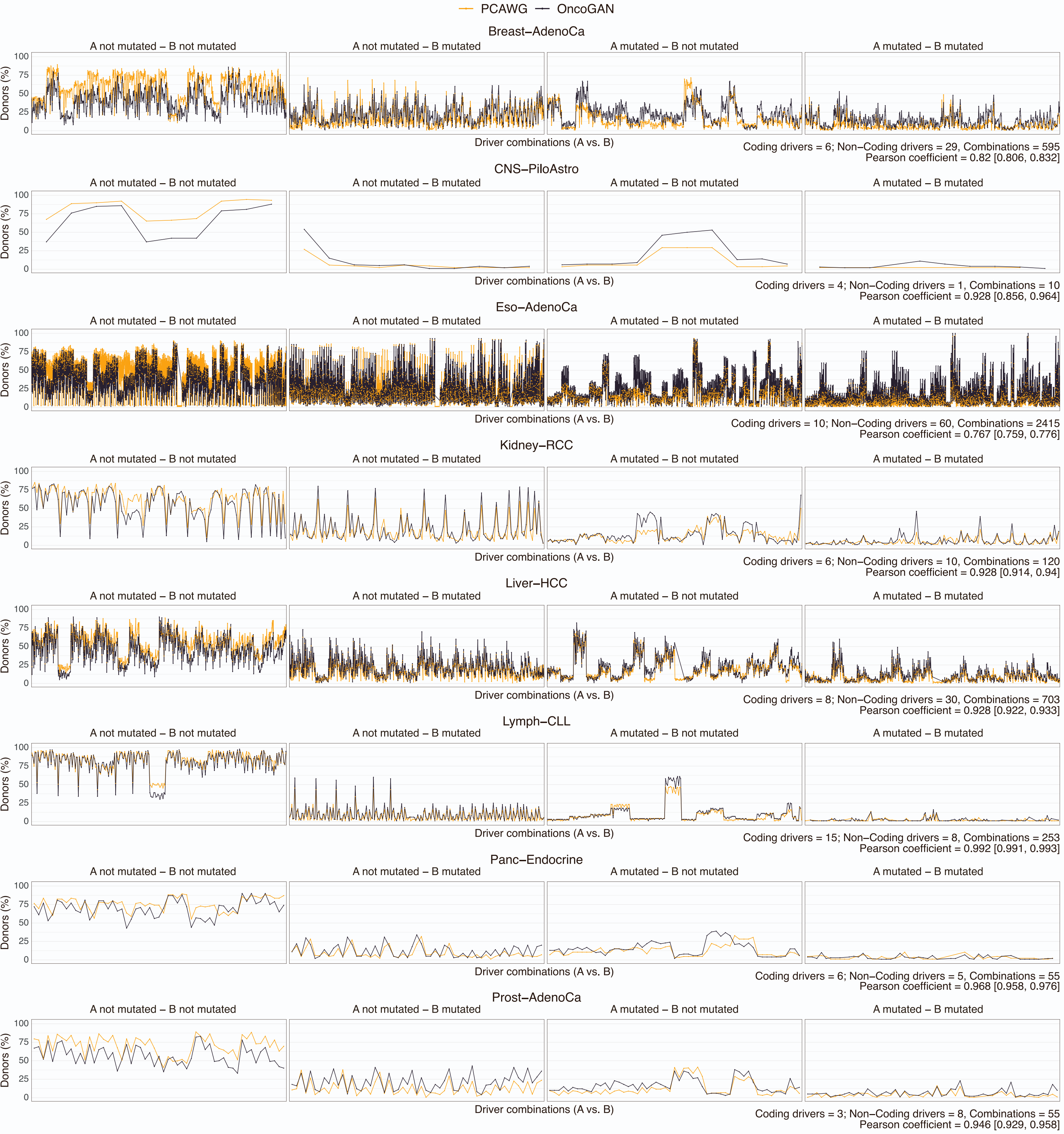

Figure S8. Driver correlation analysis plot for the seven tumor types, related to STAR Methods.
The X-axis shows all possible combinations between two driver genes, with each dot representing one 1-vs-1 combination. The Y-axis represents the percentage of donors in which that combination occurs. The number of drivers used and the total number of combinations are reported in the caption for each tumor type. The Pearson coefficient, with values exceeding 0.9 in almost all tumors, indicates a strong simulation of driver relationships. The data used to create this plot, including all possible driver combinations and their values, is listed in SuppTableS3.

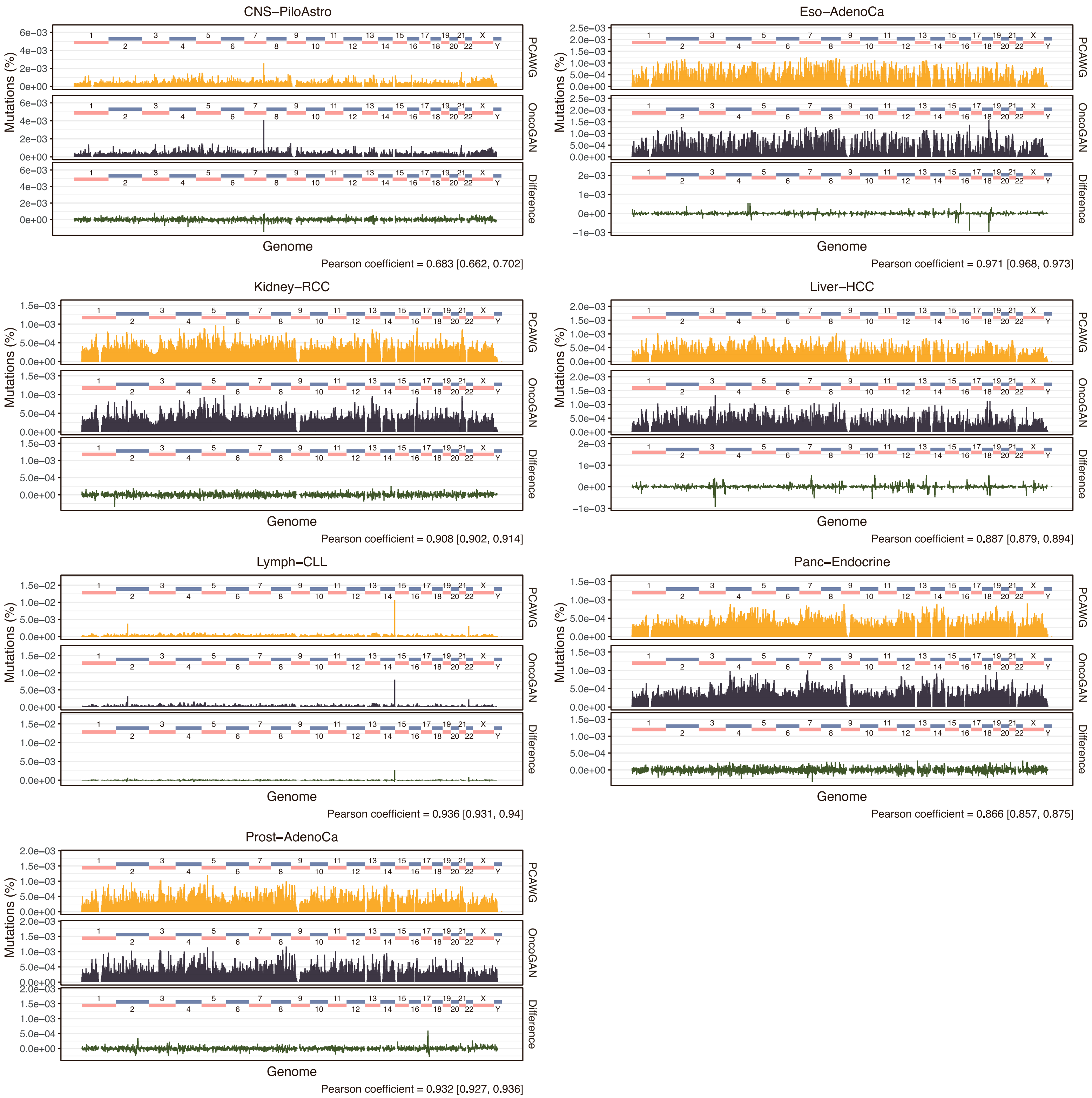

Figure S9. Histograms displaying the total percentage of mutations across the genome in 1Mbp bins for the remaining seven tumor types, related to Figure 3A.
Real donors are shown in orange, and simulated ones in black, with differences highlighted in green. Pearson correlations between PCAWG and OncoGAN genomic profiles are provided for each specific plot.

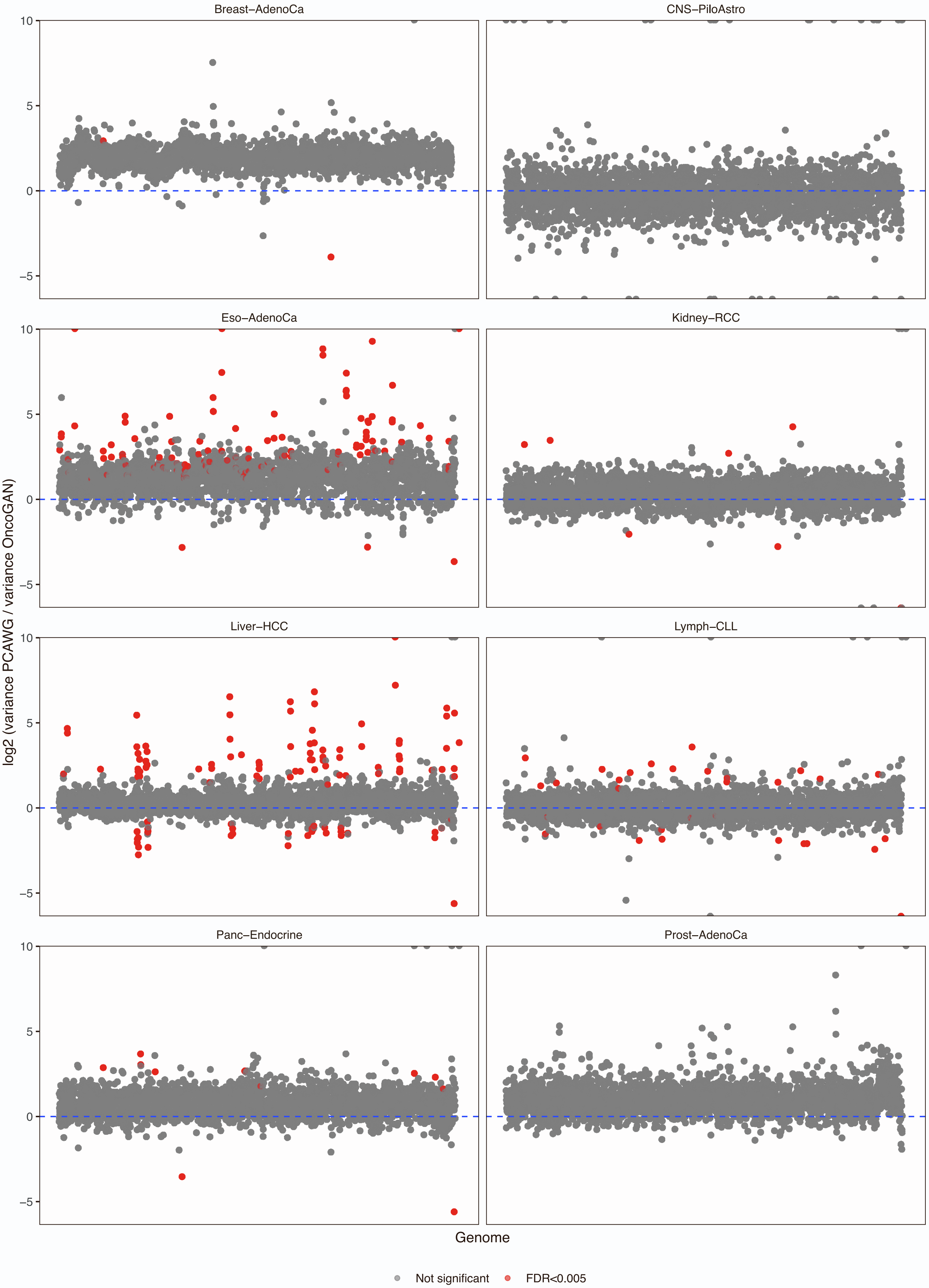

Figure S10. Analysis of mutation density variance across genomic regions in real and synthetic tumors, showing the variance ratio between PCAWG and OncoGAN donors, related to Figure 3B.
Genomic regions where variance is not well simulated are highlighted in red, indicating FDR values below 0.005.

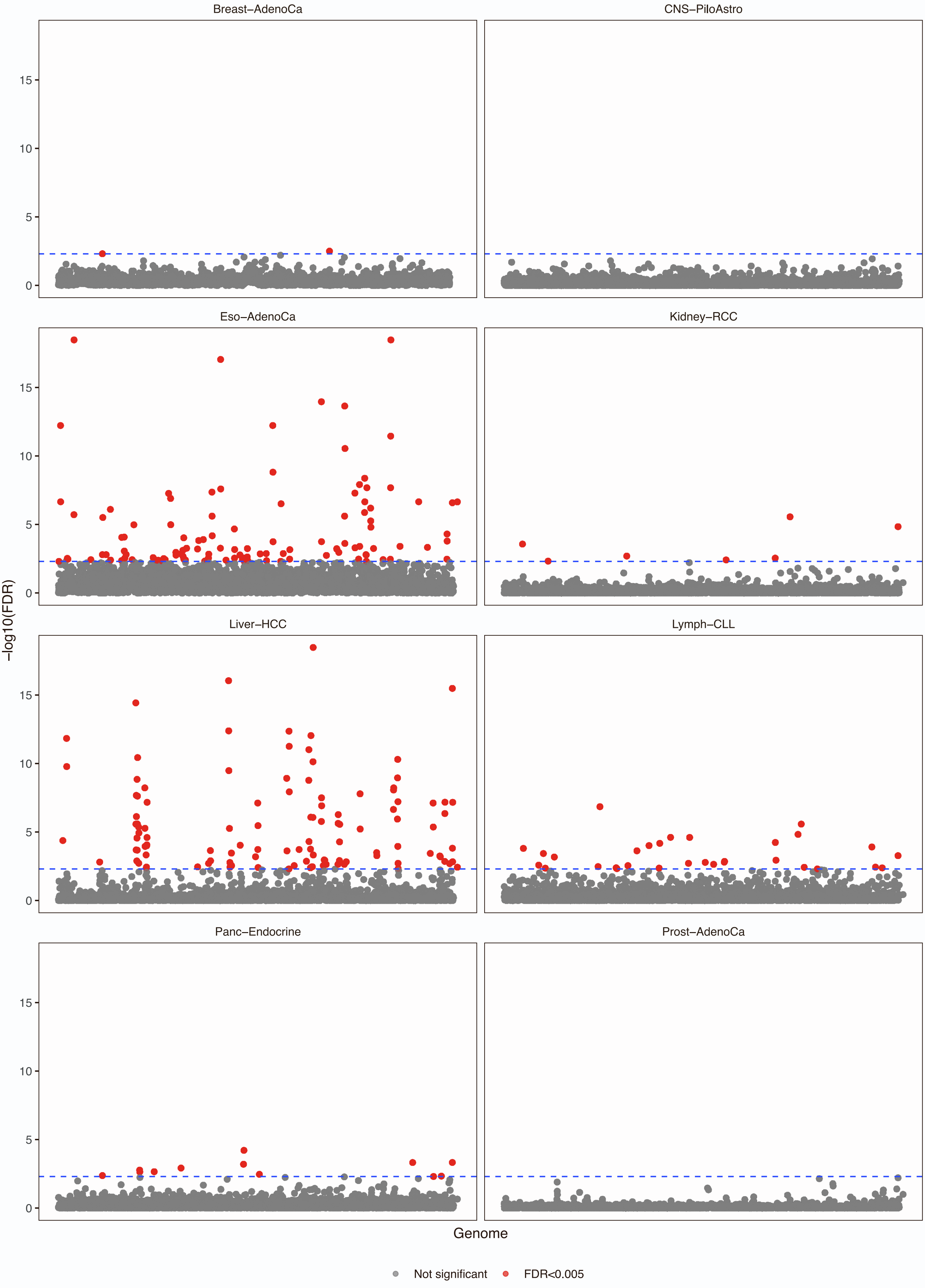

Figure S11. Manhattan plot highlighting genomic regions with significant differences in mutation density variance between PCAWG and OncoGAN donors, related to Figure 3B.

Red dots indicate regions with FDR values below 0.005.

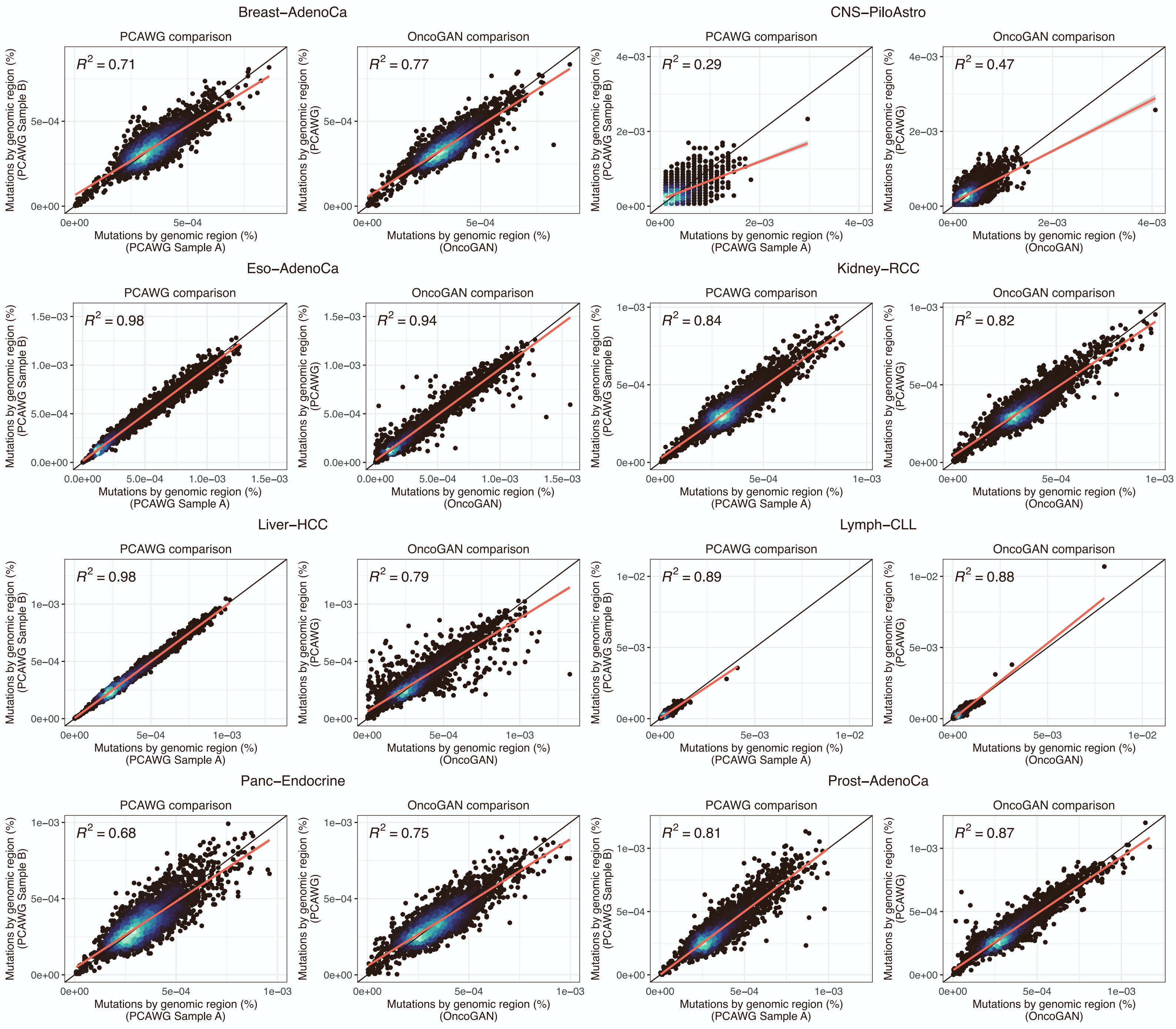

Figure S12. Scatter plot comparing mutational densities across 1Mbp genomic regions, related to Figure 3A. The comparison was made between two samples from the PCAWG dataset (left) and OncoGAN simulations against the entire PCAWG dataset (right) tumor types. Each dot represents a region, with color indicating density; lighter colors mean higher densities. R2 values are displayed for each of the comparisons.

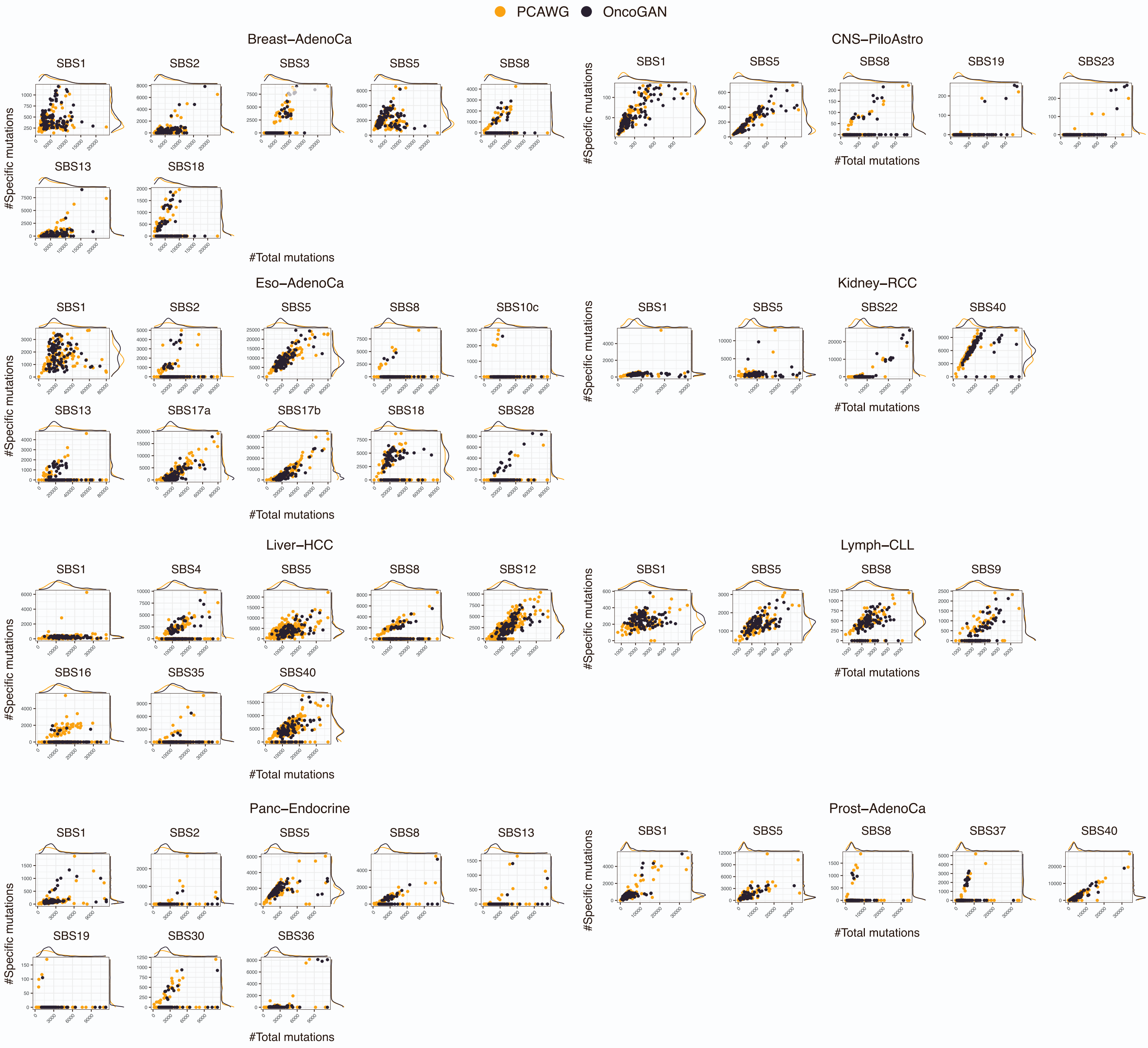

Figure S13. Scatter and density plots comparing the number of specific signature mutations to the total number of mutations for each donor, related to Figure 3C.
Real donors from PCAWG are shown in orange, and simulated donors from OncoGAN are shown in black. OncoGAN values correspond to those directly simulated by the tool.

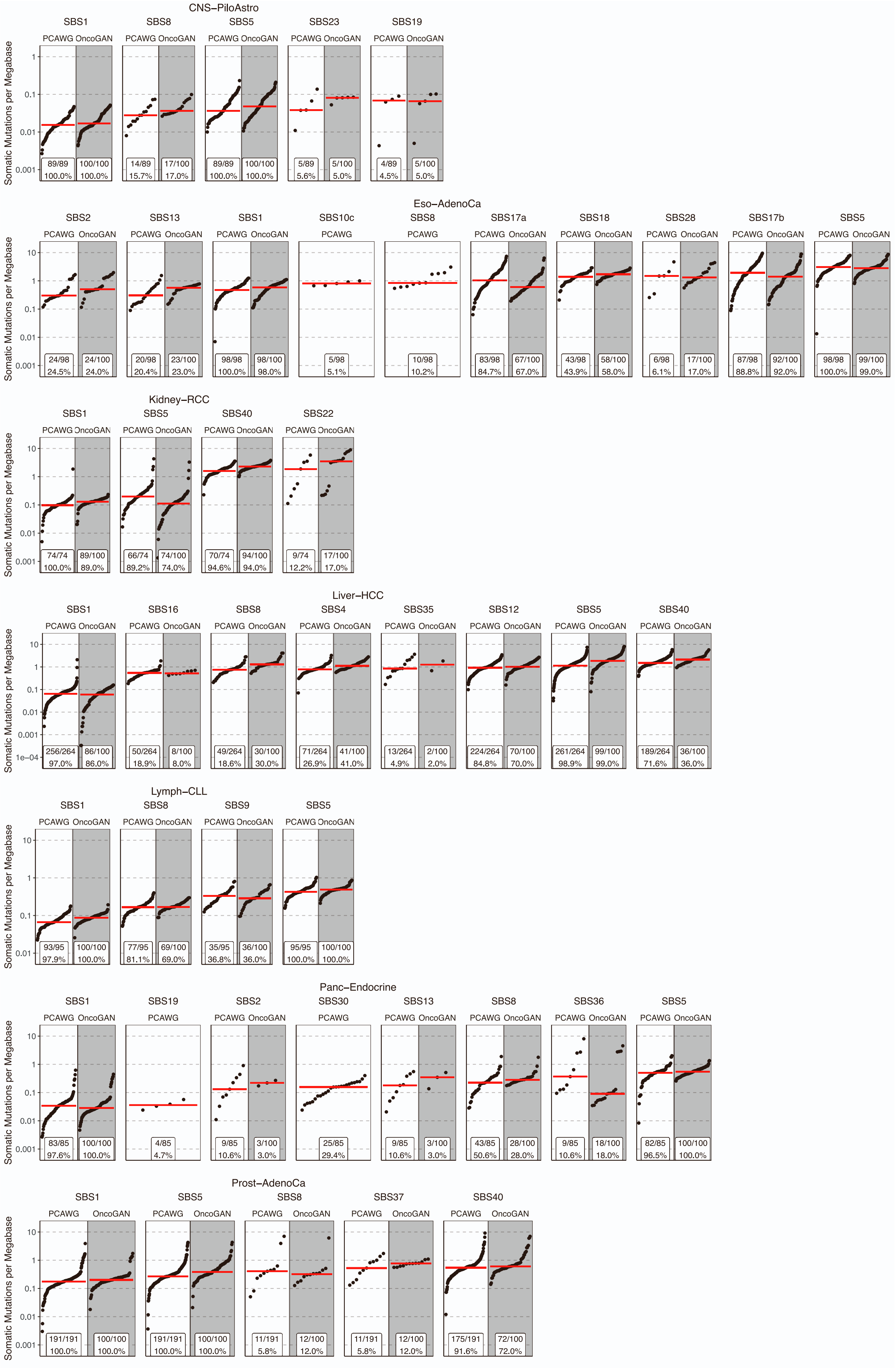

Figure S14. Mutational signatures distributions detected by SigProfiler, related to Figure 3C.
The plot shows the number of somatic mutations per megabase and the percentage of donors exhibiting each signature for the remaining tumor types. Dots represent individual donors; the red line illustrates the mean number of somatic mutations per megabase. SBS, single base substitutions.

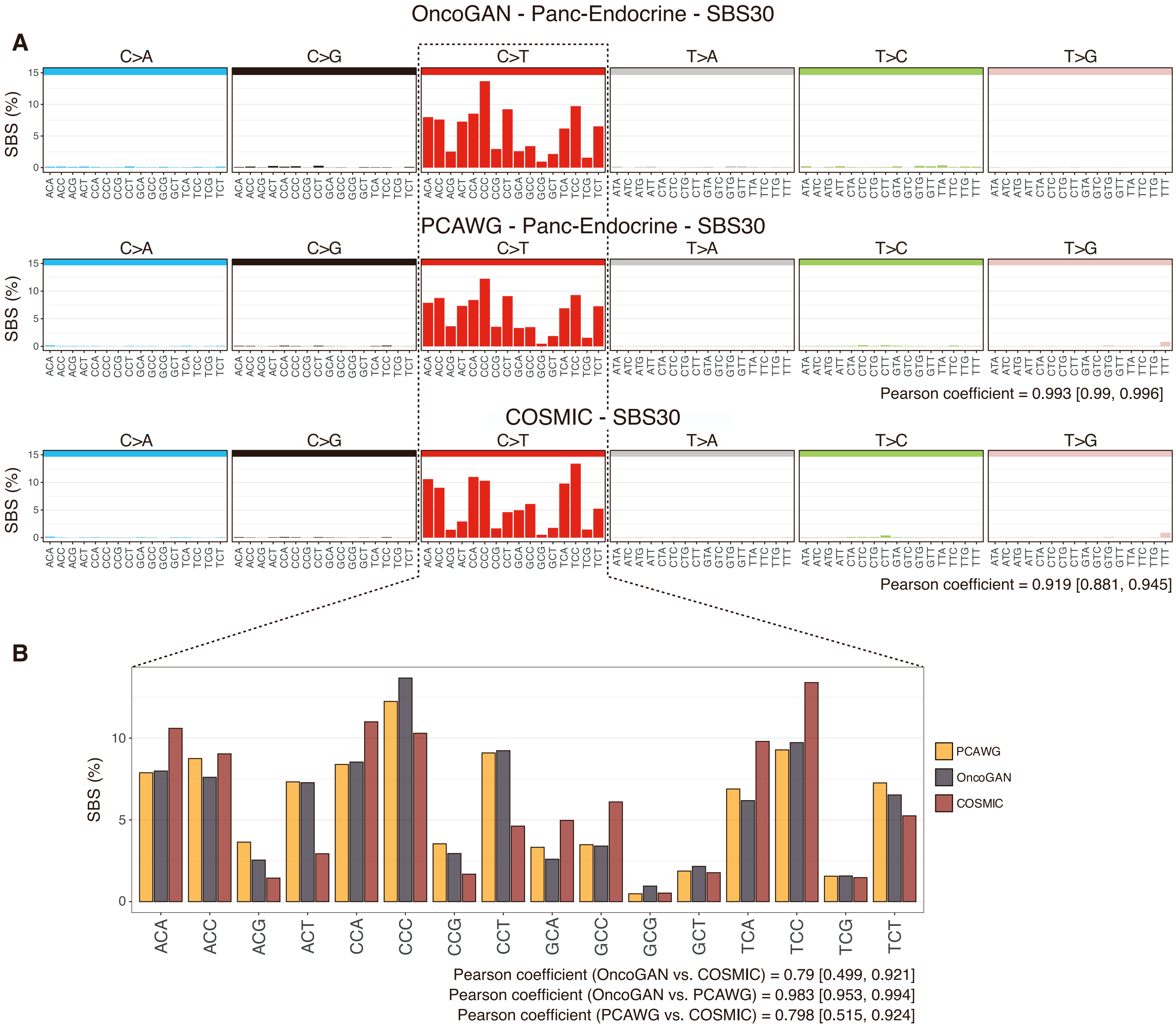

Figure S15. Comparison of the mutational pattern for the SBS30 signature in the Eso-AdenoCa tumor type across the PCAWG, OncoGAN, and COSMIC datasets, related to Figure 3C and Figure S14.
A) For all trinucleotide contexts the percentage each specific context contributes to the signature. B) Focusing on the C>T context, as it is the predominant altered context in this signature.

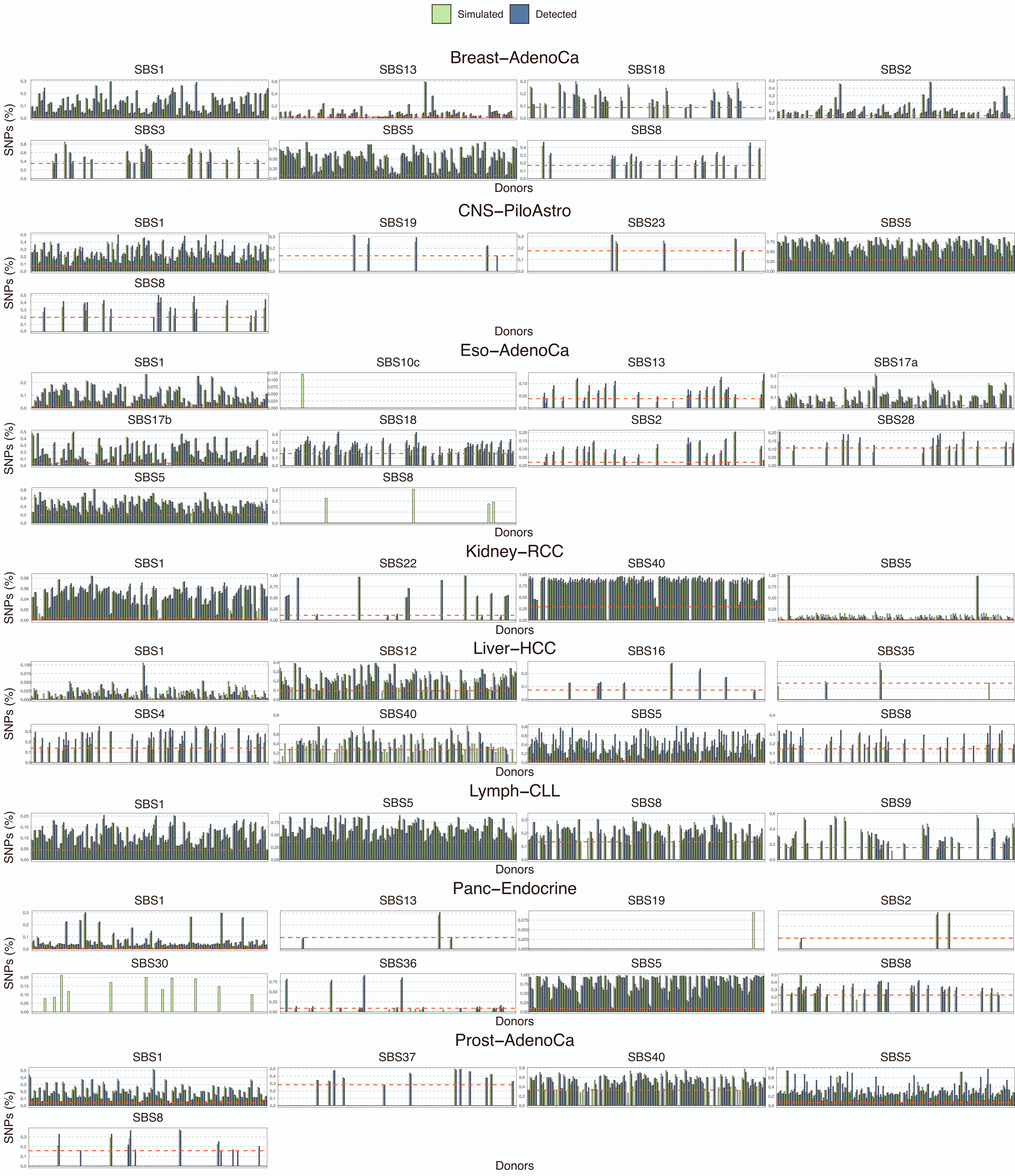

Figure S16. Comparison of the percentage of mutations directly simulated and detected for each mutational signature, related to STAR Methods.
Direct signatures simulated by OncoGAN are shown in green, whereas signatures detected by SigProfiler are shown in blue. Red dashed line indicates the lowest percentage of mutations detected by SigProfiler. SNP, Single nucleotide polymorphism.

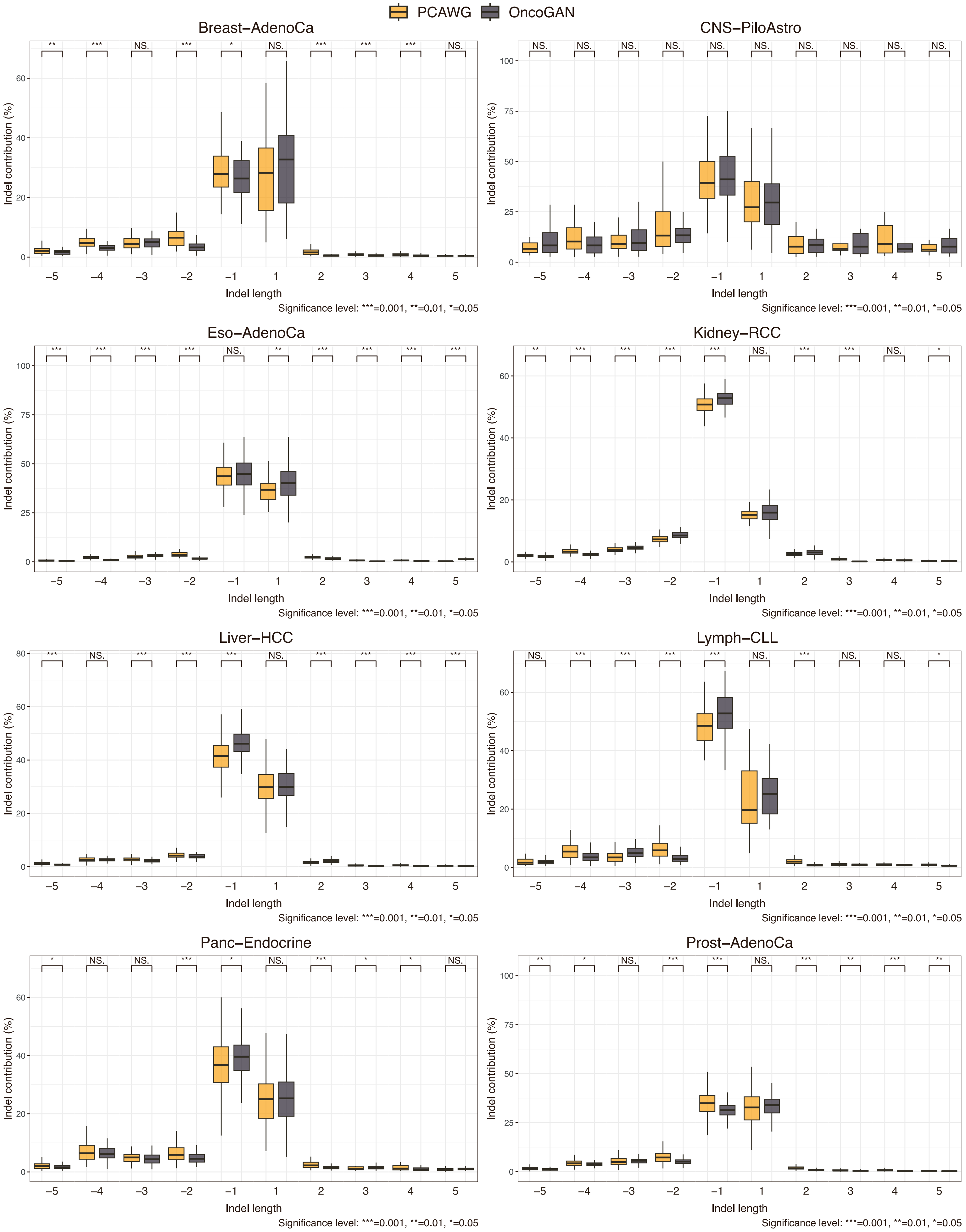

Figure S17. Boxplots comparing indel length distribution between PCAWG and OncoGAN, related to STAR Methods.

PCAWG and OncoGAN results are shown in orange and black, respectively. Negative X values represent deletions, and positive X values represent insertions. The Y-axis shows the contribution (%) of each specific indel length relative to the total number of indels per donor. Only indels up to a size of 5 are plotted. The sample size used for each comparison corresponds to the number of donors available for each tumor type. The Wilcoxon test was used to compare the groups. NS.: p-value > 0.05; *: p-value <= 0.05; **: p-value <= 0.01; ***: p-value <= 0.001.

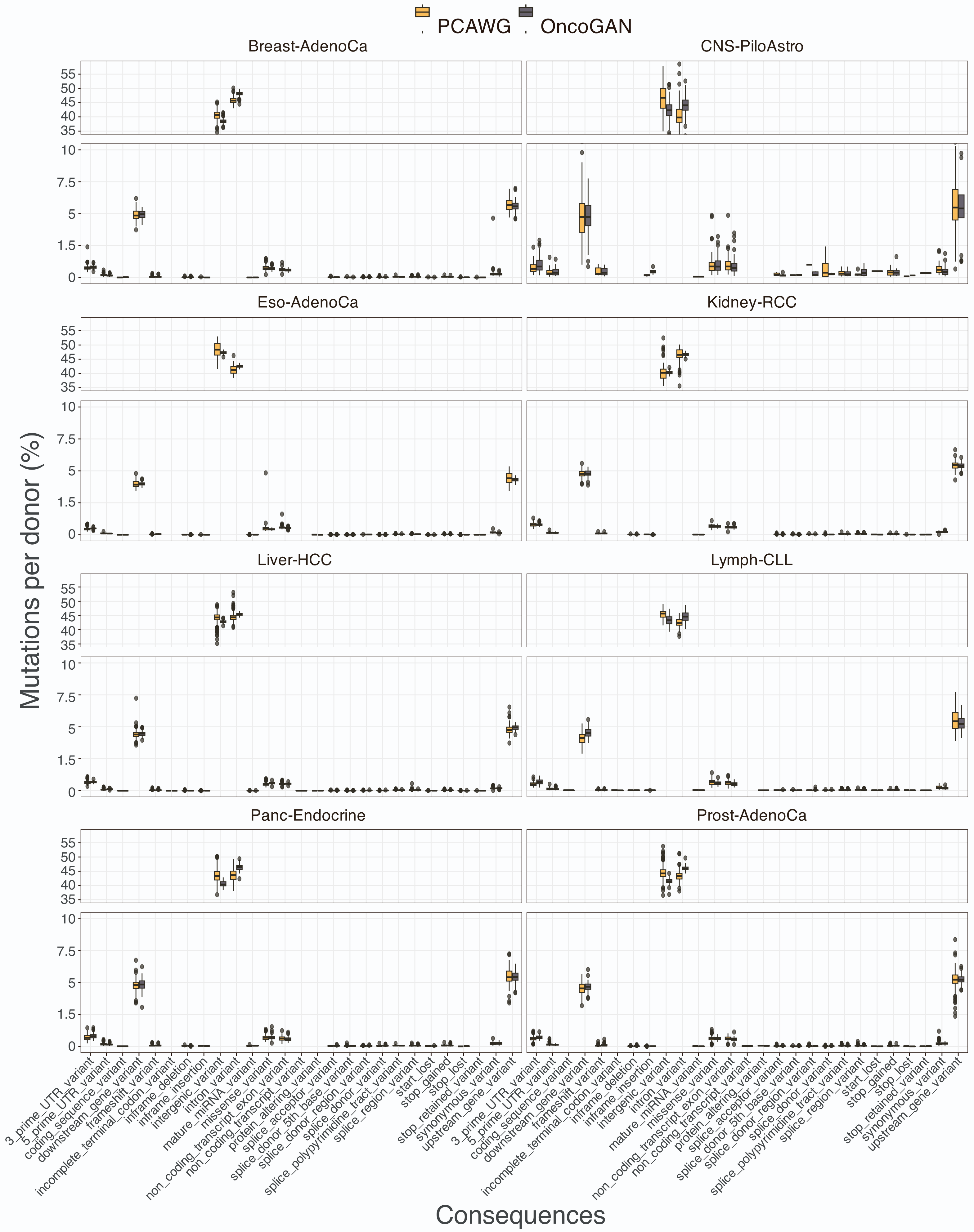

Figure S18. Boxplots comparing the frequency of predicted effects for real and simulated mutations per donor using the Variant Effect Predictor (VEP) tool, related to STAR Methods.

Real mutations are shown in orange, whereas simulated ones appear in black.The X-axis lists the possible mutation consequences considered by VEP, while the Y-axis shows the percentage of mutations corresponding to each consequence per donor.

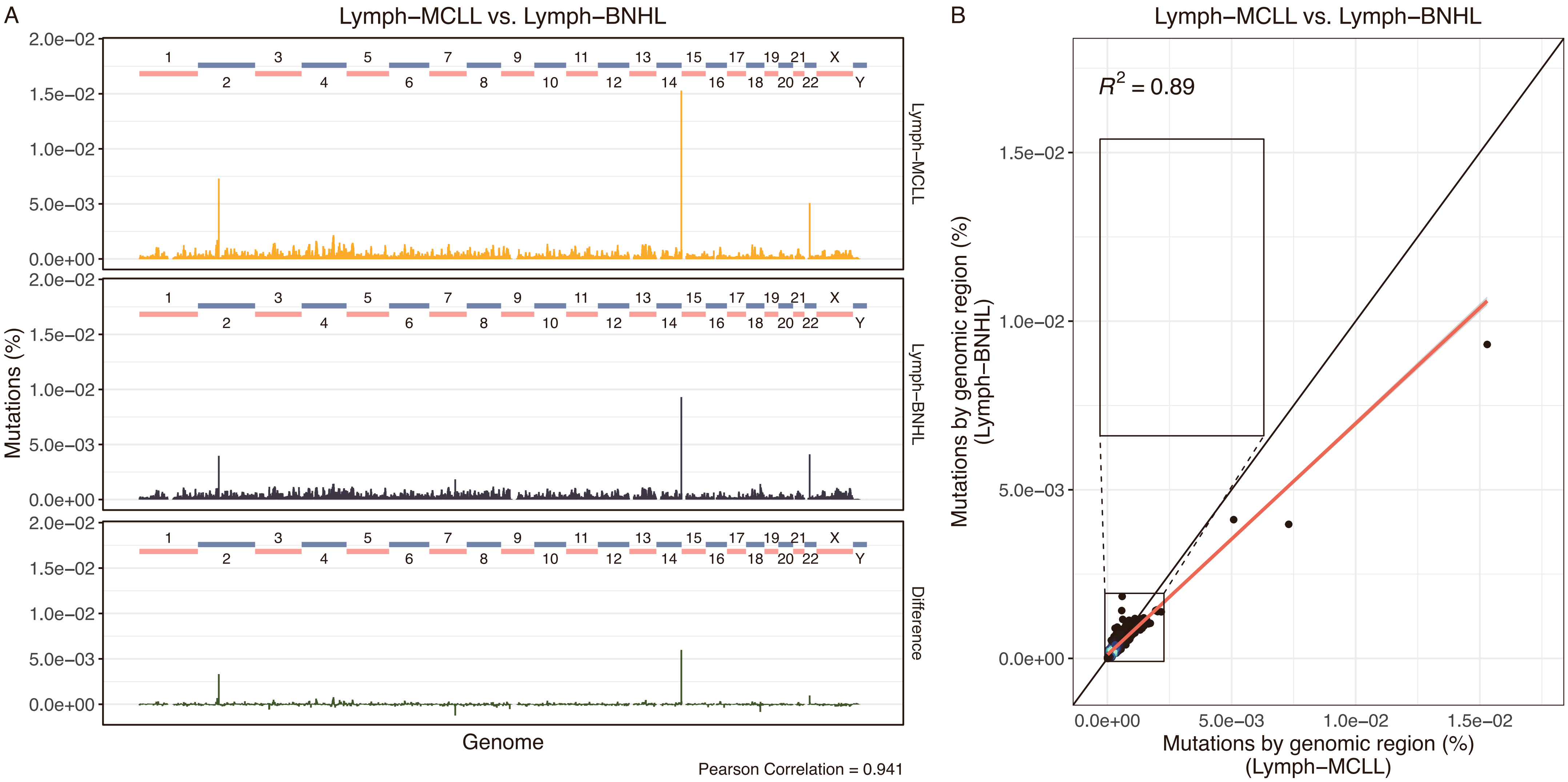

Figure S19. Comparison of real Lymph-MCLL and Lymph-BNHL tumor types illustrating their similarity in terms of genomic mutation profiles, related to Figure 3A.
A) Histogram displaying the total percentage of mutations across the genome in 1Mbp bins. Real donors are shown in orange, and simulated ones in black, with differences highlighted in green. The Pearson correlation is 0.941, indicating a high degree of similarity. B) Scatter plot comparing the percentage of mutations in each 1Mbp genomic region between both tumor types. Each dot represents a region, with color indicating density; lighter colors indicate higher densities.

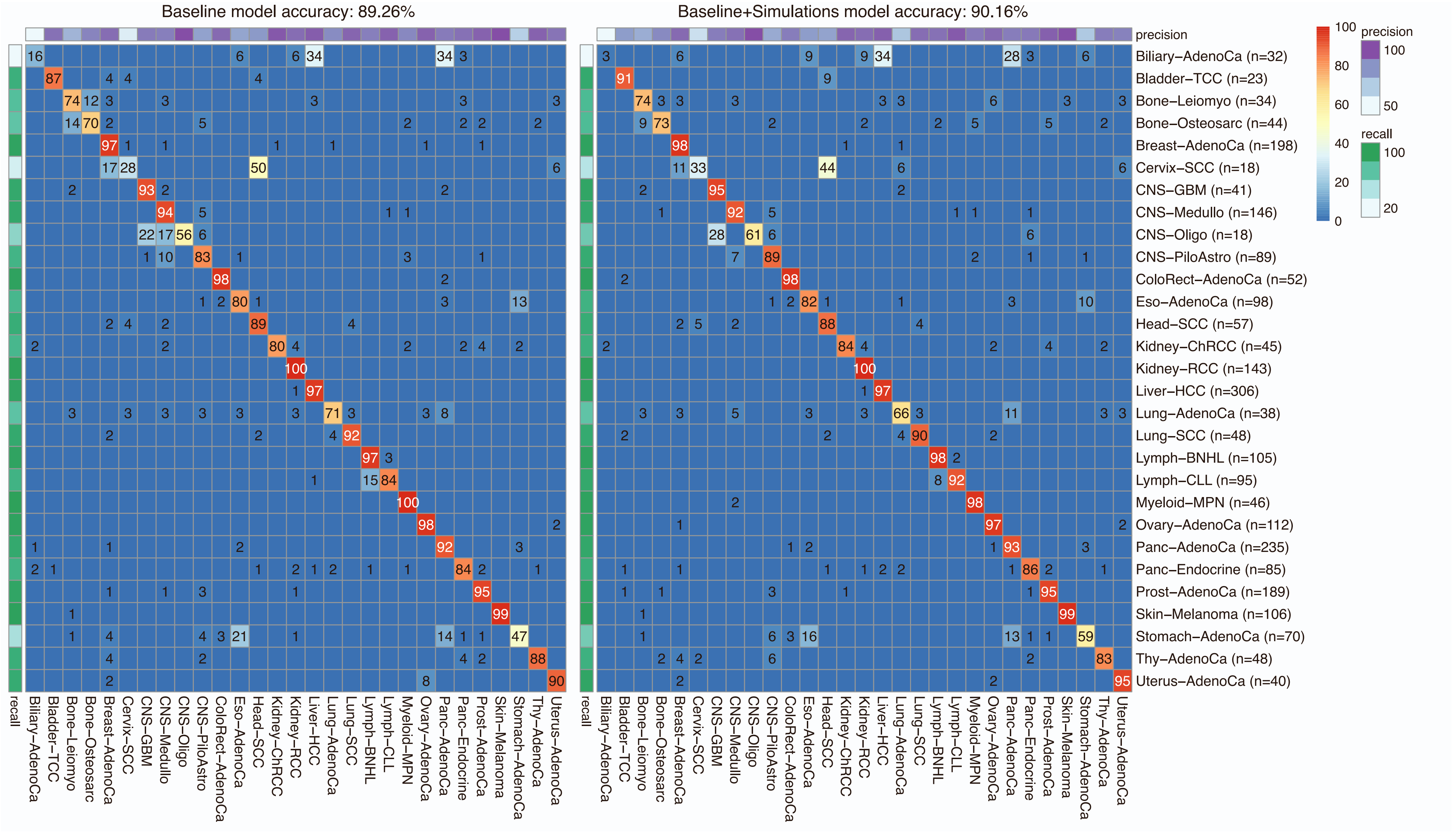

Figure S20. DeepTumour heatmap displaying the accuracy of the baseline and the new classifier using a held-out portion of the PCAWG data set for evaluation (5-fold cross-validation), related to Figure 4C.

Baseline model was trained using only PCAWG samples, whereas the new model employs a mix of real and synthetic samples. Each row corresponds to the true tumor type and columns correspond to the class predictions emitted by DeepTumour. Cells are labeled with the percentage of donors of a particular type that were classified by DeepTumour as a particular type. The recall and precision of each classifier are shown in the color bars at the top and left sides of the matrix. All values represent the mean of 5 runs using selected data set partitions.

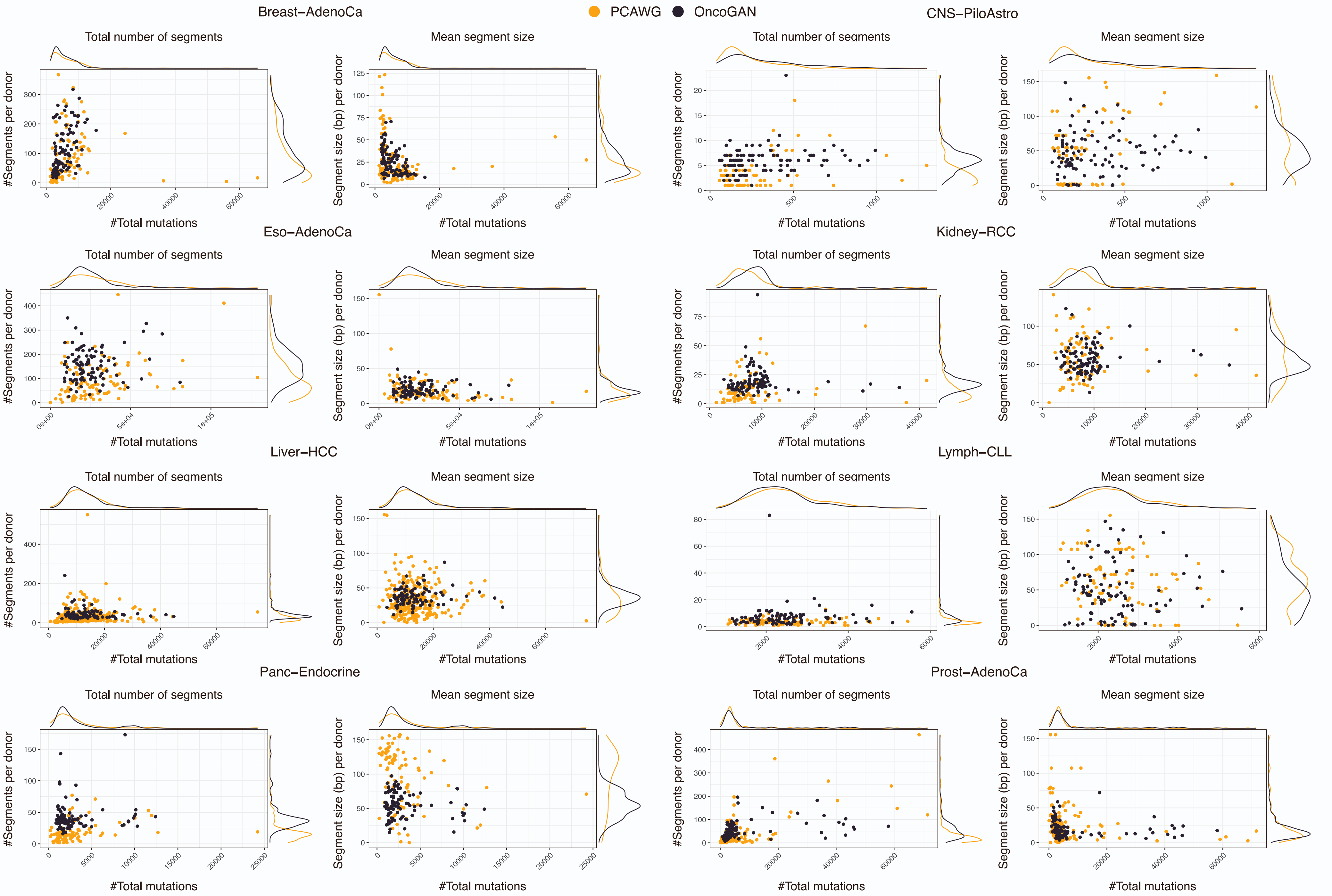

Figure S21. Scatter and density plots showing the relationship between the total number of mutations per donor and two chromosomal instability scores, related to Figure 5.

The studied scores are: the total number of aberrant segments and their mean length. As shown, the distributions of real (orange) and simulated (black) donors are highly similar.

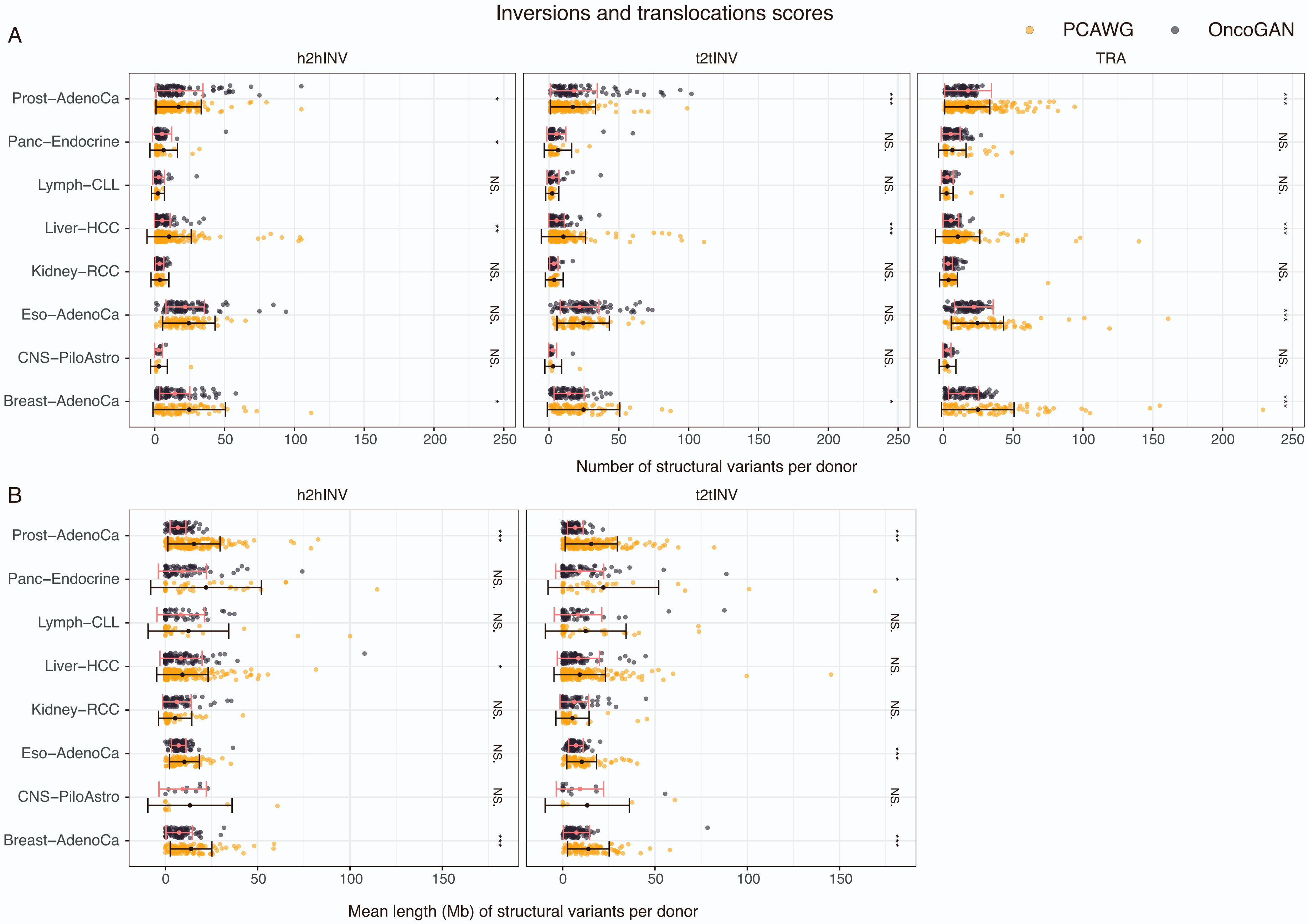

Figure S22. Chromosomal instability scores measuring the similarity of inversion and translocation SVs between real and simulated donors, related to STAR Methods.

A) Number of h2hINV, t2tINV, and TRA events per donor. B) Mean length of SVs in base pairs. Error bars represent the standard deviation of the dataset. Real and simulated donors are shown in orange and black, respectively. The sample size used for each comparison corresponds to the number of donors available for each tumor type. The Wilcoxon test was used to compare the groups. NS.: p-value > 0.05; *: p-value <= 0.05; **: p-value <= 0.01; ***: p-value <= 0.001. h2hINV, head-to-head inversion; t2tINV, tail-to-tail inversion; TRA, Translocation.
